# A Lubricating Oil Condition Monitoring System Based on Wear Particle Kinematic Analysis in Microfluid for Intelligent Aeroengine

**DOI:** 10.3390/mi12070748

**Published:** 2021-06-25

**Authors:** Zhenzhen Liu, Yan Liu, Hongfu Zuo, Han Wang, Hang Fei

**Affiliations:** 1 Civil Aviation Key Laboratory of Aircraft Health Monitoring and Intelligent Maintenance, College of Civil Aviation, Nanjing University of Aeronautics and Astronautics, Nanjing 211100, China; lzz1201@nuaa.edu.cn (Z.L.); wanghan07@nuaa.edu.cn (H.W.); feihang@nuaa.edu.cn (H.F.); 2 Center for Advanced Life Cycle Engineering, Department of Mechanical Engineering, University of Maryland, College Park, MD 20740, USA

**Keywords:** aeroengine, lubricating oil monitoring, wear particle velocity, microfluidic technology

## Abstract

Lubricating oil monitoring technology is a commonly used method in aeroengine condition monitoring, which includes particle counting technology, as well as spectral and ferrography technology in offline monitoring. However, these technologies only analyze the characteristics of wear particles and rely on physical and chemical analysis techniques to monitor the oil quality. In order to further advance offline monitoring technology, this paper explores the potential role of differences in wear particle kinematic characteristics in recognizing changes in wear particle diameter and oil viscosity. Firstly, a kinematic force analysis of the wear particles in the microfluid was carried out. Accordingly, a microfluidic channel conducive to observing the movement characteristics of particles was designed. Then, the wear particle kinematic analysis system (WKAS) was designed and fabricated. Secondly, a real-time tracking velocity measurement algorithm was developed by using the Gaussian mixture model (GMM) and the blob-tracking algorithm. Lastly, the WKAS was applied to a pin–disc tester, and the experimental results show that there is a corresponding relationship between the velocity of the particles and their diameter and the oil viscosity. Therefore, WKAS provides a new research idea for intelligent aeroengine lubricating oil monitoring technology. Future work is needed to establish a quantitative relationship between wear particle velocity and particle diameter, density, and oil viscosity.

## 1. Introduction

In recent years, aeronautical accidents have occurred frequently, with consequences such as forced landings, minor damage, crashes of the aircraft, and passenger casualties [1], showing the great importance of aircraft safety to human life. Among these accidents, aeroengine wear failure is a significant cause [2,3]. As we know, with the development of big data, 5G technology, and artificial intelligence, intelligent aeroengine has become an inevitable trend in the field of aeroengine manufacturing and maintenance [4]. The development of intelligent aeroengines is closely related to the intellectualization of the compressor, turbine, lubricating oil system, and other essential parts of the aeroengine, which all contain a large number of supporting components, such as bearings and gears. At the same time, the basis of intellectualization of the parts involves continuous and accurate working condition monitoring. However, the supporting components produce different diameters of wear particles after a long time of working, whose characteristics can reflect the mode, mechanism, and severity of wear [5]. In addition, the lubricating oil provides oil for various bearings and gears to reduce friction, and the various wear particles move along with oil in the pipeline, thereby changing the oil quality and, in turn, affecting the normal operation of all parts. Oil monitoring technology uses oil analysis as a means to monitor the wear particles carried by the aeroengine oil and the changes in the physical and chemical properties of the oil to obtain information on the engine wear status and oil decay, as well as to dynamically evaluate the engine’s working state and predict and diagnose engine failures. Therefore, the importance of finding an oil monitoring analysis system based on wear particles’ characteristics for an intelligent aeroengine cannot be overemphasized.

The technology has attracted the attention of many researchers around the world, who have studied many online analysis technologies [6], such as those based on the electrostatic principle [7], inductance principle [8,9], capacitance principle [10], and acoustic principle [11], as well as offline analysis technologies, for instance, spectral [12], ferrography [13], and optical particle analyses [14]. However, on account of the disadvantages of the harsh working environment and high noise based on the online monitoring technology, offline monitoring can provide accurate oil condition information [15]; thus, it remains the most commonly used method [3]. At present, the most frequently used technique is ferrography analysis, which involves making a ferrogram, separating the wear particles in the oil sample, washing the ferrogram, and analyzing it under a precision microscope to obtain the classification of particles. The result can reflect the type of wear between friction pairs in an engine, but it is very time-consuming and relies on the experience of the observers, while it cannot monitor the quality of the oil. At the same time, due to the direct viewing performance of optical technology and the development of precision manufacturing, OLVF, LaserNet Fines (LNF), and chipcheck122 based on optical technology have become particularly popular in the practical industry [16]. Nevertheless, these methods only analyze the characteristics of particles, and the quality monitoring of lubricating oil depends on physical and chemical analysis technology. The viscosity is the most critical quality index of lubricating oil, which is the primary parameter of lubricating oil replacement. Consequently, this paper proposes a comprehensive oil monitoring method that can integrate the wear particle property and lubricating oil kinematic viscosity. Moreover, a wear particle kinematic analysis system (WKAS) is elaborately designed and fabricated using this method, which can be applied to intelligent aeroengines.

Wear particles can be more easily controlled in the microfluidic channel compared to the macroscopic pipeline, owing to their small diameter. Accordingly, Wu et al. developed OLVF to observe the classification, recognition, and 3D reconstruction of ferromagnetic particles using algorithms in the microfluid [17,18,19,20]. In addition, Visitskii [21] depicted the settlement motion of solid spherical particles in a static viscous fluid. Khatibi [22] investigated particles of different diameters falling in rectangular water channels through particle tracking velocimetry (PTV). Ning [23] used the digital holography method to study the vertical motion characteristics of tiny spherical particles in the ocean, where it was found that the speed of particles is affected by the diameter, density, and viscosity of the fluid. However, due to the existence of boundaries and the differences in macro- and microfluids, studying the motion of wear particles in a microfluidic pipe is relatively complicated. Thus, this work aimed to study the kinematic characteristics of wear particles in a microfluidic channel filled with lubricating oil to develop a comprehensive oil monitoring method for intelligent aeroengines.

According to the above research, the force analysis of wear particles in constant flow was detailed to study their kinematic characteristics, and the size of the microfluidic channel was designed on the basis of the diameter of wear particle, since the channel plays a critical role in particle motion. Then, the hardware system of the WKAS was fabricated, primarily constituting a control system of the injection pump and a video acquisition system of moving wear particles. At the same time, the velocity and the quantity of the particles were extracted by establishing a Gaussian background mixture model (GMM) and a blob-detection algorithm, representing the main software system of WKAS. Lastly, the oil condition was monitored by integrating the diameter of the particles with the kinematic viscosity of the oil. This work provides a new research idea for aeroengine lubricating oil monitoring technology.

The rest of this paper is organized as follows: The design and the fabrication of the WKAS are presented in Section 2. Section 3 describes the experiment conducted on a pin–disc tester, and the results of oil quality monitoring are analyzed as a function of the wear particle velocity. Lastly, conclusions are drawn in Section 4.

## 2. Design and Fabrication of the WKAS

### 2.1. Theoretical Calculation of the Wear Particle Velocity

The analysis of forces on the wear particles is the first step toward determining the velocity of particles in the microchannel. Moreover, it is necessary to make some basic assumptions to form a physical model. We assumed that (1) the oil is a Newtonian fluid; (2) the concentration of particles is so low that only forces of oil on the particles are considered, whereas the forces between particles are ignored; and (3) the shape of the particle is simplified to be spherical.

**Figure 1:**
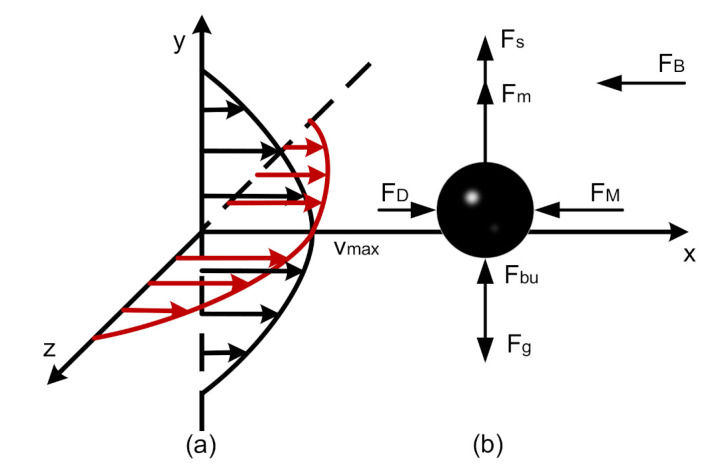
(**a**) Laminar velocity distribution: the red and black arrows indicate the velocity distribution of oil in the width and height directions of the channel, respectively, while $V_{\max}$ is the maximum velocity at the center of the channel; (**b**) various forces acting on particles in the laminar flow field (the parameters are defined in the Abbreviations).

It is generally known that the flow state of a fluid is laminar flow, as the Reynolds number in a microfluid is far less than 1 [16]. Thus, a schematic diagram of oil velocity distribution is shown in Figure 1a. Because classical Newtonian mechanics and fluid mechanics theory still apply to microfluids [24], the forces can be divided into three parts [25] according to the type of action: forces independent of the relative motion between the fluid and particles, such as gravity and buoyancy; forces that are related to the relative movement and have the same direction as the relative velocity, such as drag force, additional mass force, and Basset force, which are called generalized resistance, as shown in Equation (1); forces that are related to the relative motion and perpendicular to the relative velocity, such as Magnus force and Saffman force, which are called generalized lift, as shown in Equation (2). The details are demonstrated in Figure 1b.

Generalized resistance:

(1) $$F^{\left(s\right)}=F_{D}+F_{M}+F_{B}=3\pi \mu d_{p}(u-u_{p})+\frac{\pi }{12}d_{{}_{p}}^{3}\rho_{f}\frac{d(u-u_{p})}{\mathrm{dt}}+\frac{3}{2}d_{p}^{2}\sqrt{\pi \rho_{f}\mu }\int_{0}^{t}\frac{d(u-u_{p})/d\tau }{\sqrt{t-\tau }}$$

Generalized lift:

(2) $$F^{\left(l\right)}{=F}_{m}{+F}_{s}=\frac{\pi }{8}d_{{}_{p}}^{3}\rho_{f}{\omega (u-u}_{p})+1{.615d}_{p}^{2}\sqrt{\rho_{{}_{f}}\mu }{(u-u}_{p})\sqrt{\left|\mathrm{du}/\mathrm{dy}\right|}$$

In the microflow field, as the particles can easily contact the channel, the main forces from the boundary, including van der Waals force, electrostatic force, and modified fluid resistance, should be analyzed. However, previous experiments have indicated that their effective distances are less than 10 nm, 100 nm, and 1 μm, respectively [26,27,28]. Therefore, this paper did not consider these three confined forces. Similarly, not all the forces mentioned above have the same order of magnitude. Simplifying the model was the key to obtaining reasonable results. Since drag force plays a significant role in laminar flow, the acting forces on the particle were compared with the drag force, as shown in Table 1.

In this working environment, as an example, for the “MOBIL LUBRICANT 0W-40” oil with ϑ = 69.87 mm^2^/s, $\rho_{f}$ = 850 kg/m^3^, diameter of particles = 50–100 μm [17], only 1.7–6.7 × 10^5^ s^−1^ Magnus lift can reach the same order of magnitude as the drag force. Magnus lift is ignored because the rotational angular velocity of wear particles is impossible to achieve in a microfluid. In the same way, the Saffman force is also negligible due to the velocity gradient (du/dy) of the oil being hard to reach (2.7 × 10^5^) when the particle diameter is 100 μm. In addition, the relative velocity between the fluid and the particles gradually remains constant under the conditions of steady microflow, whereby $d(u-u_{p})/dt=0$; thus, the additional mass force and Basset force also do not need to be considered.

According to the above analysis, it can be realized that the drag force, gravity, and buoyancy should be taken into account in this theoretical model. Hence, the particle remains suspended when the density of the particle is close to or less than that of the oil. Otherwise, it is inclined to sediment toward the bottom of the channel. Moreover, the particle comes to a steady state quickly, whose velocity is equal to zero in the *y*-direction owing to the height of the microchannel typically being short. Nevertheless, the particle moves at a constant velocity under the driving force of oil in the *x*-direction in both circumstances; therefore, this paper mainly considered the horizontal drag force. According to the theory-of-force analysis, the motion equation for moving particles can be written as follows:

(3) $$\rho_{p}.\frac{4}{3}\pi {\left(\frac{d_{p}}{2}\right)}^{3}.\frac{du_{p}}{dt}=3\pi \mu d_{p}(u-u_{p})$$

This equation is a first-order nonhomogeneous differential equation, which, after solving, yields the following:

(4) $$u_{p}=\left(1-e^{-\frac{18\mu }{\rho_{p}d_{p}^{2}}t}\right)u$$

It can be known that, theoretically, the velocity of particles in the *x*-direction has a certain relationship with the dynamic viscosity of the oil, the particle diameter, etc. Thus, it is feasible to explore the viscosity of the oil and the diameter of the particles as a function of the velocity of the particles to monitor the oil quality for intelligent aeroengines.

### 2.2. Design of Microfluidic Channel

**Figure 2:**
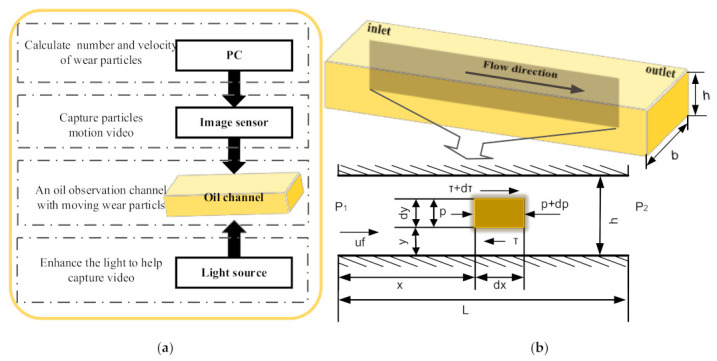
(**a**) Principle of the WKAS; (**b**) flow analysis of oil in the microfluidic channel.

In general, the motion capture of wear particles based on image technology includes a light source, oil observation channel, and motion-image capture sensor, as exhibited in Figure 2a. In such a structure, the oil channel is a crucial component as moving particles are imaged, and the channel size is closely related to the oil velocity. Considering that a circular pipe is not conducive to observing particles, which are naturally refracted by light, a micro-rectangular channel composed of four pieces of glass was designed. As illustrated in Figure 2b, the width, height, and length of the flow channel were set as b, h, and L, respectively. In order to study the relationship between oil velocity and the size of the channel, a microelement body dx/dy in the flowing oil was analyzed to list the force-balance equation.

(5) pdy+(τ+dτ)dx=(p+dp)dy+τdx

where $\tau =\mu \frac{du}{dy},\frac{dp}{dx}=\frac{p_{2}-p_{1}}{l}=-\frac{\Delta p}{l}$. Then, after double integration, we obtain the following:

(6) $$u=\frac{1}{2\mu }\frac{dp}{dx}y^{2}+C_{1}y+C_{2}$$

where the boundary conditions exist when y = 0, u = 0, and y = h, u = 0; therefore, the rate of flow is as follows:

(7) $$q=\int_{0}^{h}-\frac{y}{2\mu }(h-y)\frac{dp}{dx}bdy=-\frac{bh^{3}}{12\mu }\frac{dp}{dx}$$

It can be seen that q is proportional to h^3^, indicating that the height of the flow channel has a significant influence on the oil flow rate, which must be strictly designed. At the same time, in order to accurately capture the particle’s motion video, a higher oil velocity would result in a higher camera frame rate, thus increasing the hardware cost. Peng pointed out that the maximum size of wear particles is about 150 μm before equipment is damaged [16]. Consequently, the depth of the microfluidic channel was set to 200 μm to avoid blocking the channel. Moreover, the width was set to 300 μm to decrease the range of motion of particles.

In addition, referring to the results in Reference [16], we see that the entrance length of the laminar fluid was taken as 100D = 100 × 2bh/(b + h) = 2.4 cm [29]; hence, the length was set to 3 cm, allowing the moving particles to be observed at a distance of 2.5 cm from the entrance. In summary, the length, width, and height of the microfluidic channel were set to 3 cm, 300 μm, and 200 μm, respectively. Attention should be paid to keeping the channel surface clean to prevent noise in digital-image sequences.

### 2.3. Fabrication of the WKAS Hardware System

**Figure 3:**
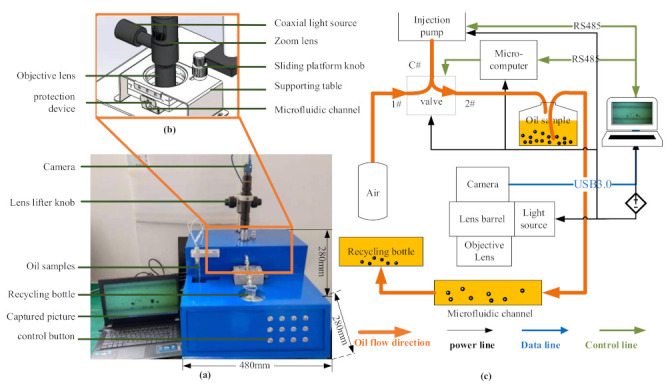
(**a**) Complete hardware system of WKAS, (**b**) partial assembly diagram of microfluidic channel, and (**c**) WKAS working principle diagram.

The model of the WKAS was designed by using SolidWorks software, whose length, width, and height were 480, 280, and 280 mm, respectively. The finished product after matching (All Controller Aviation Technology Co., Ltd., Nanjing, China) is shown in Figure 3a. In consideration of the light transmittance of common materials, the observation channel was made of quartz glass, and an iron protection device was fixed on the outside, as shown in Figure 3b. Meanwhile, a sliding platform was equipped on top of the protection device, enabling the channel to move smoothly with a precision of 0.01 mm; as such, the particles at any position in the *x*-direction would be visible. Meanwhile, the platform was fixed on the inner base of a supporting table. In addition, it was necessary to combine an objective lens and zoom lens with a magnifying function to capture the particle images, due to their sizes typically being below 150 μm [17]. Because the lens had a limited depth of field, a precise lens lifter was employed to adjust the focal distance of the zoom lens, enabling the particles at any location in the *y*-direction to be observed. Table 2 provides the details of the apparatus. The software StreamPix was used for video and image acquisition, allowing the adjustment of acquisition duration and exposure settings, as well as the export of image sequences in various formats. The motion video of wear particles in the microfluidic channel can be captured at a rate of 125 fps. Furthermore, an injection pump and a high-pressure valve were introduced to achieve precise control of the oil velocity. Lastly, the function of the microcomputer was to control the opening and closing of each valve interface.

The working principle diagram of the WKAS is illustrated in Figure 3c. The image sequences and the computer data were transmitted through a USB 3.0 data line. Moreover, the control module of the injection pump and the three-way valve used an RS-485 interface to communicate with the computer. The power voltage used in the experiment was 220 V/50 Hz or 24 V DC. To begin, the pump entered suction mode, and air entered the pump from path 1 and path C of the three-way valve. Then, path 1 was closed, path 2 was opened, and the pump entered the push model. The oil sample began to flow under air pressure. The diameter of the pipeline was 1.5 mm, and it was made of Teflon with strong corrosion resistance. Under the illumination of the zoom lens barrel’s coaxial light source, the particle motion video was captured by the camera and stored on the computer when the particles passed through the microfluidic channel. Eventually, the oil with wear particles entered the recycling bottle.

### 2.4. Particle Tracking and Velocity Measurement Algorithm

**Figure 4:**
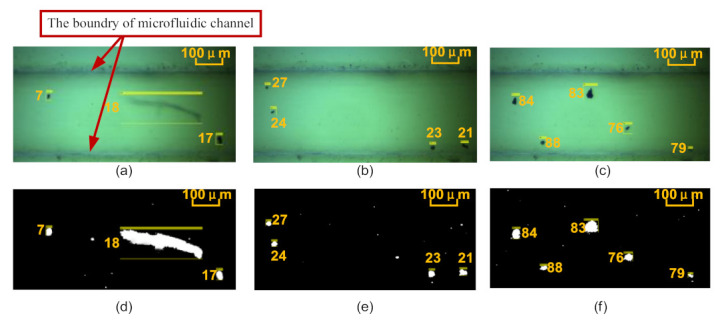
Example of automatic tracking results for wear particles: (**a**) original image of particles #7, #17, and #18; (**b**) original image of particles #21, #23, #24, and #27; (**c**) original image of particles #76, #79, #83, #84, and #88; (**d**) binary image of particles #7, #17, and #18; (**e**) binary image of particles #21, #23, #24, and #27; (**f**) binary image of particles #76, #79, #83, #84, and #88.

**Figure 5:**
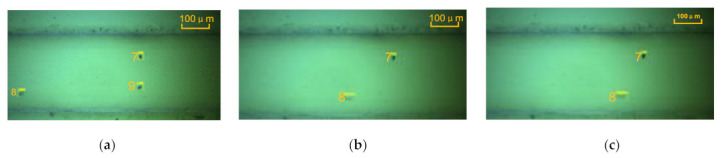
Images of particles #7 and #8: (**a**) frame #1, (**b**) frame #5, and (**c**) frame #6.

The wear particle detection, tracking, and velocity calculation algorithms were developed through the Gaussian mixture model (GMM) [30] and blob algorithm in this study. Figure 4 shows some example of automatic tracking results for wear particles. We can see that all wear particles could be tracked accurately by using boxes of different sizes. Furthermore, the number, the transverse and longitudinal coordinates of the particles, the frame ID of the images, the length and height of the tracking box, the area (the projection surface of the particle), and the equivalent circle diameter were extracted. Each particle, regardless of size and shape (strip, round, oval, and irregular), had a unique ID number. Yellow rectangular boxes automatically matching the size of the particle, such as particles #7, #17, #18, #21, #23, #24, #27, #76, #79, #83, #84, and #88, were tracked in turn, and the tracking results show that the algorithm has high robustness. The particle’s velocity could be calculated by dividing the pixel position by the time of the particle appearing in the observation area and leaving. It can be seen in Figure 5 that, although the sizes of particles #7 and #8 were similar, the movement distance of particle #8 and its velocity were greater upon tracking frames #1, #5, and #7. The reason is that particle #8 was suspended in the oil and moved forward, whereas particle #7 rolled along in the microchannel, due to their different densities. This indicates that particles could be tracked and identified in real time regardless of their motion characteristics, thus verifying that the algorithm based on GMM and blob could be effectively used for measuring particle velocity.

## 3. Experimental Section

### 3.1. Experimental Setup

**Figure 6:**
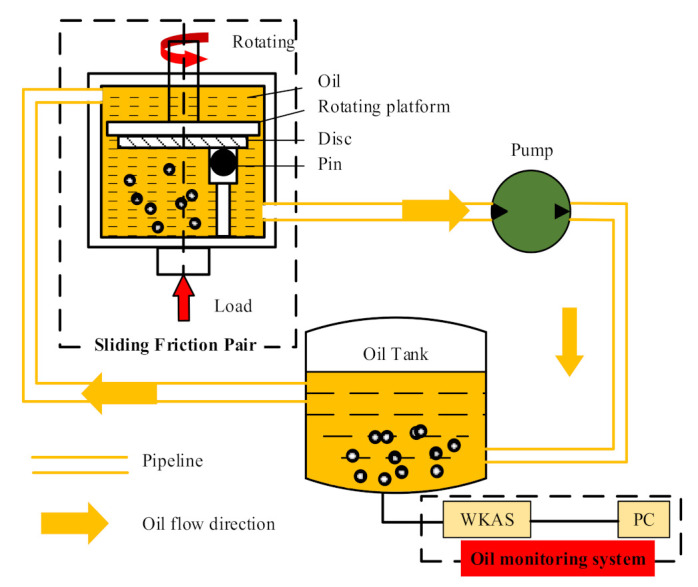
Functional diagram of offline oil monitoring experiment on a pin–disc test rig.

In this section, we detail how oil condition monitoring based on wear particle kinematic analysis in a microfluid was carried out by using the WKAS and procedures described in Section 2. For this purpose, a pin-on-disc test rig was established. The experiment was performed in the Key Laboratory of Civil Aviation Aircraft Health Monitoring and Intelligent Maintenance at the Nanjing University of Aeronautics and Astronautics. Figure 6 displays the experiment equipment and functional diagram. To efficiently simulate the wear process of most rotating parts in an aeroengine, a sliding friction pair was built composed of a disc and a pin. Rotation of the disc was achieved by mounting it with a rotating platform, using bolts, whereas a vertical load was exerted on the pin by a bottom spring device, while fixed by a support rod. Table 3 lists the parameters of the pin and disc.

Then, the wear particles were generated from the sliding friction pair. In order to efficiently collect wear particles, the oil supply port and the oil return port were installed at an angle of 270° to the rotating platform. An electric motor was used to drive the peristaltic pump to absorb oil from the oil tank, and the moving oil drove the particles to the tank. A constant load of 80 N was applied in the experiment, and the flow rate of oil was 1 m/s. After the test rig stopped working, a 10 mL oil sample was collected from the oil tank. It should be noted that the oil sampling was completed within 30 min of the equipment being shut down, and the room temperature was maintained at 20°, thereby ensuring that the viscosity of the oil was not affected. Subsequently, a 10 s video of the moving particles was acquired using the WKAS when the oil flow state became stable. Next, morphology feature extraction and velocity calculation of the wear particles were carried out using the tracking algorithm proposed in Section 2. Accordingly, the wear state and oil condition of the test rig were comprehensively determined by using these parameters to realize the oil condition monitoring of an intelligent aeroengine.

### 3.2. Results and Discussion

It is widely known that, as the severity of mechanical equipment wear increases, the diameter of the wear particles will gradually become larger, the number of them will also increase, and the oil viscosity will become lower. To evaluate the performances of the WKAS, three experiments were carried out. The one was to examine the ability to monitor the particle diameter. The two was to examine the ability to monitor the oil kinematic viscosity. The last was to verify that particles of the same diameter move at different velocities in different oil, and the fixed velocities of particles could provide the viscosity of lubricating oil.

#### 3.2.1. Monitoring of Particle Diameter Based on Average Velocity in the Lubricating Oil

**Figure 7:**
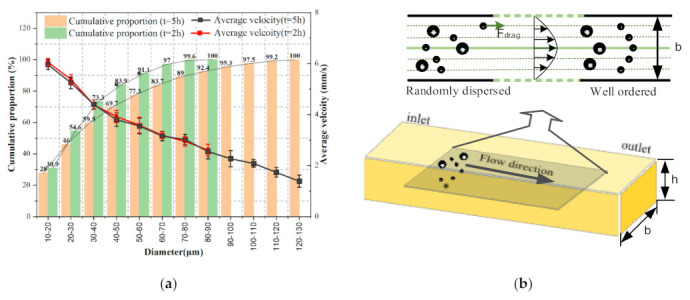
(**a**) Average velocity of wear particles and cumulative proportion of particles with different diameters at different working moments; and error bar = SD, (N = 3). (**b**) Motion diagram of particles with different diameters in the microflow pipe.

Considering that wear particle diameter monitoring is a crucial part of oil monitoring, which can describe the wear severity of equipment, average velocity was picked to estimate the changes in diameter of wear particles during this process. Hence, 0W-40 lubricating oil was selected as the experimental oil, and the speed grade of the pump was set to 1. Thus, the average flow rate of the oil in the microfluidic channel was 10 mm/s after calculation. When the working time of test rig reached 2 and 5 h, three oil samples were collected, respectively. It can be seen from the WKAS that particles with different diameters moved along the straight line at different speeds in the *x*-direction, and the motion states included suspension, rolling, and sliding. Figure 7a shows the proportion of particles with different diameters and the changes in wear particle average velocity as a function of the diameter at different working moments. The two columns chart guide us to the conclusion that with the extension of working hours, the severity of equipment wears increases, and the number of large-diameter particles increases. We can also find that the velocity of particles in the same diameter range was almost identical, and the maximum average speed was 6 mm/s, which conforms to the force analysis of particles presented in Section 2.1. That is, after the oil movement was stable, the particles moved along the flow direction at a velocity lower than that of the oil.

In addition, the velocity of particles correlated with their diameter, whereby larger diameters resulted in a lower average particle velocity. The reason is that drag force plays a key role in determining the movement of particles in the laminar flow field, which is closely related to the diameter. Thus, the particles showed better followability to the oil when their diameter was smaller. Given that the velocity curve of laminar flow is parabolic, as the particle entrance neared the center of the channel, the velocity became greater. Hence, the instantaneous velocity of particles with the same diameter was also different. However, due to the small width of the microfluidic channel, larger particle diameters typically resulted in a lower average velocity, as shown in Figure 7b.

**Figure 8:**
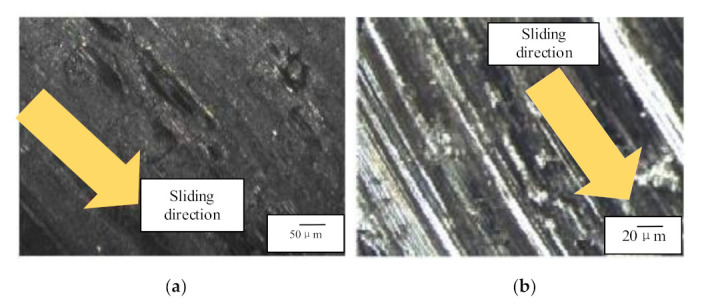
Surface microscopic images: (**a**) pin and (**b**) disc.

Meanwhile, it can be seen in Figure 7a that particles with a diameter less than 50 μm accounted for almost 70% of all particles when the working time was 5 h, while the largest diameter recorded was 130 μm. Using the results from Reference [17], it can be obtained that the pin and disc experienced a run-in period, a stable wear period, and a severe wear period. Figure 8 shows the surface images of the pin and disc after the experiment, using a parallel stereo microscope. It can be seen that the friction pair exhibited severe sliding wear since the disc had obvious adhesive pits and shallow scratches, with the extrusion deformation in its sliding direction, while the pin also had clear grooves. Therefore, the average velocity of wear particles could be extracted to determine their diameter in the oil, thereby allowing the equipment’s wear state to be monitored through the developed WKAS.

#### 3.2.2. Monitoring of Lubricating Oil Kinematic Viscosity Based on Average Particle Velocity

**Figure 9:**
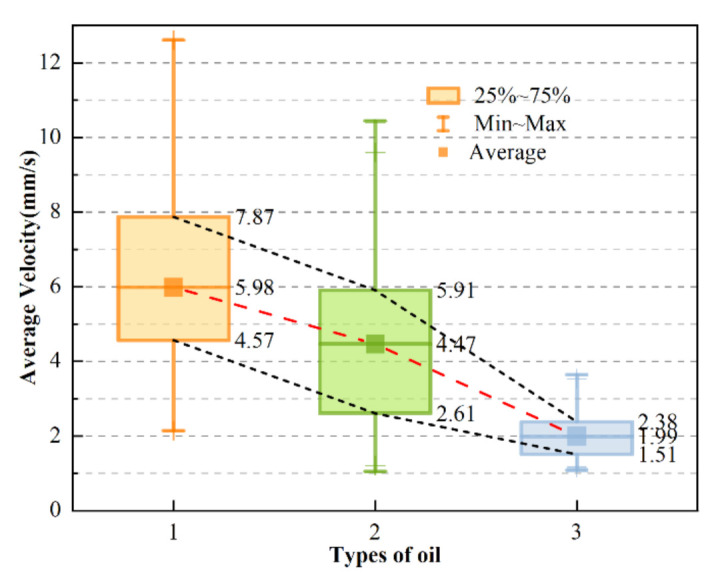
Box plot showing the average velocity of particles in three oils of different viscosity.

Another important aspect of oil condition monitoring is the kinematic viscosity of the oil. Accordingly, three commonly used oils with kinematic viscosities of 36.06, 69.87, and 165.2 mm^2^/s were picked for study, as measured by the Spike ultra-Q3000 portable kinematic viscometer, and they were named No. 1, No. 2, and No. 3. Then, an experiment was successively carried out in triplicate, according to the steps in Section 3.1, using the same operating mode. The difference is that the speed grade of the pump was set to 2 in order not to block the microfluidic channel, where it was calculated that the average flow rate of the oil in the microfluidic channel was 20 mm/s. The average particle velocity could then be obtained, as shown in Figure 9.

It can be seen that the average particle velocities were 5.98, 4.47, and 1.99 mm/s in the three types of oil, respectively. Thus, it could be concluded that, as the oil viscosity increased, the average velocity of all particles generally showed a downward trend. Moreover, the upper and lower quartiles of particle velocity in the three types of oil revealed the same trend. We can conclude from Equation (7) that greater oil viscosity results in a lower oil flow rate. Combined with Equation (4), it can be seen that a lower oil flow rate results in lower particle movement speed, indicating that oil viscosity can be predicted as a function of the trend in average particle velocity.

#### 3.2.3. Establishing Oil Viscosity Measurement Curve Based on Velocities of Particles with Fixed Diameter

**Figure 10:**
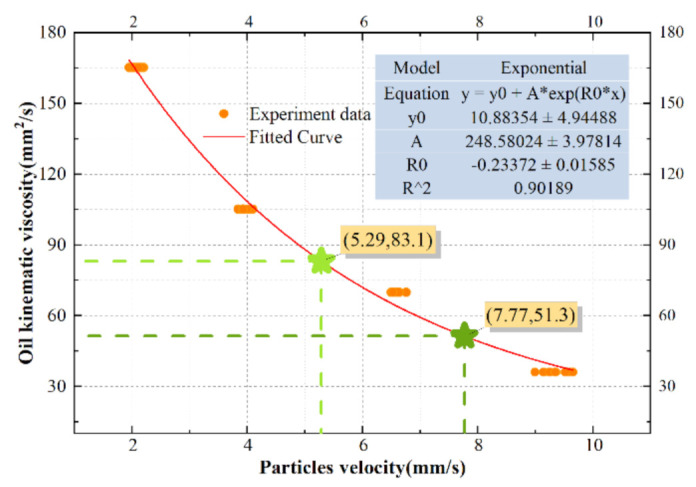
Oil viscosity measurement curve based on nonlinear exponential regression equation.

To verify that the WKAS is able to monitor the oil condition, oil viscosity measurement curve was established based on nonlinear regression equation in Figure 10. A total of 40 samples of experimental data were collected in this section; they are composed of four groups of motion experiments of particles with a diameter of 50 μm in oils with four different viscosities, as shown in Table 4. Where, the names of the oils are MOBIL LUBRICANT 0W-20, 0W-40, 15W-40, and 20W-50. The particles with diameter of 50 μm were common in the wear process of aeroengine [17], and their dimensions could be calculated from images taken by the camera. They were purchased from Yingtai Metal Materials Co., Ltd. (Zhejiang, China). At the same time, they were selected through a standard sieve in this experiment. As their shapes were irregular, three significant digits were reserved after the decimal point. The average flow rate of the oil in the WKAS was 20 mm/s. Then the velocities of particles could be obtained through the algorithm in Section 2.4. The exponential function was used to fit all data points. R^2^ is an indicator, which can reflect the degree of fit between the estimated value of the fitted curve and the actual value of the experiment.

(8) $$R^{2}=1-\frac{\sum_{i}{\left(y_{i}-f_{i}\right)}^{2}}{\sum_{i}{\left(y_{i}-\bar{y}\right)}^{2}}$$

where 1≤i≤40; $y_{i}$ is the value of the experimental data, and each is associated with a fitted value $f_{i}$; and $\bar{y}$ is the mean of all experimental data. Thus, the closer the value of R^2^ is to 1, the higher the reliability of the curve. It can be seen from Figure 10 that R^2^ was 0.90189, indicating a good degree of fitting. Moreover, the fitting-curve equation was as follows:

(9) $$y=10.88+248.58e^{-0.23x}$$

where x and y are the velocity of particle with a diameter of 50 μm and oil kinematic viscosity, respectively.

Finally, two oils with different viscosities were used to verify the effectiveness of the fitting curve. In the two sets of experiments under the same working conditions, the speeds of particles with a diameter of 50 μm wear are 5.29 and 7.77 mm/s, separately. The viscosity values of the fitted curve should be 83.1 and 51.3 mm^2^/s. Furthermore, the values measured by the Spike ultraQ3000 portable kinematic viscometer were 81.12 and 50.07 mm^2^/s, thus verifying that the oil viscosity measurement curve based on particle velocity was relatively accurate.

The above experiments show that WKAS based on the kinematic characteristics of wear particles can not only obtain the particle size as a traditional particle analyzer and ferrography analyzer, but also provide multidimensional information such as the viscosity of oil. In the future, we can use WKAS to simultaneously obtain values of oil viscosity and wear particle properties, so as to achieve the purpose of using one instrument to dynamically monitor changes in oil quality and engine wear, thereby greatly broadening the applications of oil monitoring technology.

## 4. Conclusions

In this study, a lubricating oil condition monitoring system based on WKAS for intelligent aeroengines was proposed and fabricated. The WKAS consisted of an injection pump, high-pressure valve, camera, objective lens, light source, microcomputer, and microfluidic channel, as well as particle tracking and velocity measurement algorithms. The primary conclusions are outlined below.

(1) The influencing factors of wear particle velocity were systematically studied. It was found that the wear particle velocity is closely related to the diameter of the particle and the viscosity. Furthermore, the effect of microfluidic channel size on the oil flow rate was analyzed, which was optimized as 3 cm × 300 μm × 200 μm.
(2) In view of the wear particle motion image characteristics, a particle velocity measurement algorithm based on GMM and blob was developed, and the tracking results showed that the algorithm has high robustness.
(3) As demonstrated experimentally, WKAS can be used to characterize the changes in particle diameter and oil viscosity. A larger particle diameter or greater oil viscosity results in lower average particle velocity. Notably, compared with other traditional oil condition monitoring methods, WKAS not only provides multidimensional oil condition monitoring information but also reflects wear severity. Thus, it is obvious that WKAS has great potential application in the condition monitoring of intelligent aeroengines.

This study showed that a method based on the analysis of wear particle velocity is feasible in the practical application of lubricating oil monitoring, and it has certain industrial application value, thus greatly improving oil monitoring technology. Furthermore, after improving the camera configuration, lens parameters, chip technology, etc., of WKAS, it can be used for online monitoring. Future work will include using a large number of orthogonal experiments to study the quantitative relationships among parameters with the greatest impact on the motion characteristics of wear particles (e.g., particle material, particle diameter, and lubricating oil viscosity) and the particle velocity, as well as establishing an empirical formula for oil monitoring based on the WKAS to improve the dimension and accuracy of oil monitoring technology, provide more insights into the future development of this field, and predict the failure of aeroengines in advance. In addition, a theoretical analysis of the instantaneous velocity of irregular-shaped particles based on WKAS is also needed in the future.

## Figures and Tables

**Table 1:** The ratio of the forces acting on the particles to the drag force.

${F/F}_{D}$	Ratio
$F_{D}{/F}_{D}$	1
$F_{m}{/F}_{D}$	$\omega d_{p}^{2}/24\vartheta$
$F_{s}{/F}_{D}$	$0.16d_{p}\sqrt{\left|du/dy\right|/\vartheta }$
$F_{M}{/F}_{D}$	$d_{p}^{2}\rho_{f}d(u-u_{p})/36\mu (u-u_{p})dt$
$F_{B}{/F}_{D}$	$\frac{d_{p}}{2(u-u_{p})}\sqrt{\frac{\rho_{f}}{\pi \mu }}\int_{0}^{t}\frac{d(u-u_{p})/d\tau }{\sqrt{t-\tau }}$

**Table 2:** Details of the apparatus used in this study.

Name	Corporation	Model
Microchannel	Huarui Chip Technology Co., Ltd., Zhenjiang, China	Quartz glass
Slide platform	Super Eye Technology Co., Ltd., Shenzhen, China	Z006
Objective lens/lens lifter	Jiangnan Novel Optics Co., Ltd., Nanjing, China	10X/SHL-10A
Zoom lens barrel	Shanghai Optical Co., Ltd., Shanghai, China	2×
Camera/video capture software	Daheng Imaging Co., Ltd., Beijing, China	MER2-301-125U3C/StreamPix
Injection pump/valve	Runze Fluid Co., Ltd., Nanjing, China	MiNi-SY04/Mrv-01B
Microcomputer	All Controller Aviation Technology Co., Ltd., Nanjing, China	MC9S08DZ128

**Table 3:** Parameters of the pin and disc.

Test Piece	Material	Ingredient	Size/mm	Surface Roughness (μm)	Surface Hardness (HRC)
Pin	GCr15	1.01% C; 1.50% Cr; 0.30% Mn; 0.25% Si; ≤0.02% S; ≤0.027% P	ϕ6	0.04	60
Disc	GCr15	1.01% C; 1.50% Cr; 0.30% Mn; 0.25% Si; ≤0.02% S; ≤0.027% P	ϕ80 × 8	0.4	10

**Table 4:** Experimental data of particles move at different velocities in different oil.

Run	Diameter (μm)	Velocity (mm/s)	Viscosity (mm^2^/s)
1	49.252	9.613	36.06
	49.381	9.548	36.06
	49.674	9.604	36.06
	50.246	9.142	36.06
	50.678	9.354	36.06
	51.054	9.523	36.06
	48.263	9.648	36.06
	49.947	9.249	36.06
	49.324	9.345	36.06
	50.126	8.994	36.06
2	50.134	6.813	69.87
	50.249	6.799	69.87
	50.631	6.802	69.87
	50.247	6.854	69.87
	49.958	6.792	69.87
	50.854	6.757	69.87
	49.594	6.838	69.87
	49.246	6.743	69.87
	48.937	6.817	69.87
	50.133	6.728	69.87
3	50.168	4.256	105.1
	49.624	4.195	105.1
	51.029	4.213	105.1
	50.847	4.152	105.1
	49.581	4.094	105.1
	49.874	3.996	105.1
	48.249	3.958	105.1
	50.319	3.942	105.1
	50.197	4.013	105.1
	51.273	3.934	105.1
4	50.156	2.199	165.2
	51.217	2.056	165.2
	51.349	2.183	165.2
	50.487	2.158	165.2
	50.149	2.059	165.2
	49.638	1.953	165.2
	49.826	1.995	165.2
	49.659	2.037	165.2
	49.593	2.105	165.2
	50.164	2.112	165.2

## Data Availability

Not applicable.

## Abbreviations

**Symbol**	**Unit**	**Description**
b	μm	Width of the microfluidic channel
h	μm	Height of the microfluidic channel
L	μm	Length of the microfluidic channel
D	μm	Equivalent diameter of the microfluidic channel
p	${N/m}^{2}$	Oil pressure
τ	${N/m}^{2}$	Shear stress
q	$m^{3}/s$	Oil flow
$d_{p}$	μm	Wear particle size
Re	/	Reynolds number
$\rho_{f}$	${\mathrm{kg}/m}^{3}$	Oil density
$\rho_{p}$	${\mathrm{kg}/m}^{3}$	Particle density
μ	kg·m/s	Oil dynamic viscosity coefficient
ϑ	$m^{2}/s$	Oil kinematic viscosity coefficient
u	m/s	Oil instantaneous velocity
$\bar{u}$	m/s	Oil average velocity
$u_{p}$	m/s	Particle velocity
t	s	Time
g	${m/s}^{2}$	Acceleration of gravity
$F_{g}$	N	Gravity
$F_{\mathrm{bu}}$	N	Buoyancy
$F_{D}$	N	Drag force
$F_{M}$	N	Additional mass force
$F_{B}$	N	Basset force
$F_{m}$	N	Magnus force
$F_{s}$	N	Saffman force
